# Patients’ Continuing Use of an Online Health Record: A Quantitative Evaluation of 14,000 Patient Years of Access Data

**DOI:** 10.2196/jmir.3371

**Published:** 2014-10-24

**Authors:** Richard G Phelps, Joanne Taylor, Keith Simpson, Jasmine Samuel, A Neil Turner

**Affiliations:** ^1^University of EdinburghRenal Autoimmunity GroupCentre for Inflammation ResearchEdinburghUnited Kingdom; ^2^Glasgow Royal InfirmaryGlasgowUnited Kingdom

**Keywords:** electronic health records, patient access to personal records, chronic renal insufficiency, utilization

## Abstract

**Background:**

Online access to all or part of their health records is widely demanded by patients and, where provided in form of patient portals, has been substantially used by at least subgroups of patients, particularly those with chronic disease. However, little is reported regarding the longer-term patient use of patient-accessible electronic health record services, which is important in allocating resources. Renal PatientView (RPV) is an established system that gives patients with chronic kidney disease access to live test results and information about their condition and treatment. It is available in most UK renal units with up to 75% of particular patient groups registered in some centers. We have analyzed patient use out to 4 years and investigated factors associated with more persistent use.

**Objective:**

Our aim was to investigate RPV use by patients over time from initial registration in order to understand which patients choose to access RPV and the endurance of its appeal for different patient groups.

**Methods:**

We analyzed an anonymized extract of the database underlying RPV containing information on patient registration and events including patient access and the arrival of new blood test results or letters that patients might wish to view.

**Results:**

At the time of the extract, there were 11,352 patients registered on RPV for 0-42 months (median 17). More than half of registrants became persistent users, logging in a median of 2.0 times each month over post-registration intervals of up to 42 months (median 18.9). Provision of assistance with first logon was strongly associated with becoming a persistent user, even at 3 years. Logons by persistent users occurred around the time of consultations/tests, strongly suggestive of patient engagement. While indices indicative of greater deprivation were the strongest determinants of non-participation, they had negligible influence on drop-out rates among established users.

**Conclusions:**

In this mature patient portal system, a large proportion of patients made regular use of their online health records over protracted periods. The patterns and timing of use indicate strong patient interest in detailed information such as recent test results and clinic letters. Supporting patients through the first steps of establishing access to their online records is associated with much higher rates of long-term use of RPV and likely would increase use of other electronic health records provided for patients with chronic disease.

## Introduction

Patient-accessible electronic health records (EHRs) could have many advantages, but patient enthusiasm and use have been variable [1,2]. The more successful systems have provided information that patients or relatives need, usually in the setting of chronic disease [3-8], high criticality [9], or pertinence [10]. Variation in uptake and use has been related to social factors, and concerns have been expressed about a digital divide [11-15] and the cost in relation to utilization and health outcomes [16]. While patient use of EHRs has been evaluated in several studies, patients’ continuing use over longer terms has not been reported.

Renal diseases are mostly chronic conditions and sometimes progressive, so dialysis or renal transplantation is required. Patients with progressive or end-stage renal disease are under regular specialist supervision for the rest of their lives. They may therefore have a strong incentive to become interested in the monitoring and treatment of their condition. In particular, blood tests change in important ways in kidney disease and are typically undertaken every 3-12 months in CKD patients and patients with stable transplants, and monthly in patients receiving center-based hemodialysis.

Renal PatientView (RPV) was established in 2004 to provide information to patients with chronic kidney disease (CKD) (see the Renal PatientView website for a demo [17]). RPV enables patients to see their unscreened blood test results, doctor’s letters, information links, and certain other health data using a standard Web browser on any computer. There is no financial barrier or incentive to patients’ use of the system. Renal units make small annual payments for access. Patients are made aware of RPV in various ways including during consultations and by local advertising. Interested patients sign a request for their health data to be sent from their treatment center to RPV and then receive the link to RPV, a user name, and password with which to access RPV from a Web browser. The first time patients log on to RPV, they are required to change their password. Every logon is recorded so it is possible to determine when and for how long patients choose to continue their use of RPV.

Patient and staff responses to RPV have been quite positive (in the case of staff, this is despite common early hesitancy) [6,18]. RPV has been offered to patients of an increasing proportion of renal units in the United Kingdom since 2005, and by mid-2013 it was implemented in 60 of the United Kingdom’s 73 renal units.

In order to better understand variation in RPV uptake and persistence of usage, we analyzed the registration and access log data from the RPV server log files collected over nearly 4 years.

## Methods

### Description of the Datasets

RPV data extant on September 7, 2009 (the RPV census date), were stripped of patient identifiers and transferred to a research database. Subsets of the data were extracted to R for statistical analysis (the R Foundation for Statistical Computing [19]).

The dataset comprised patient factors listed in Table 1. Deprivation scores were obtained by searching patients’ postal codes against the Index of Multiple Deprivation (IMD; available for postal codes in England and Wales) and Scottish Index of Multiple Deprivation (SIMD; available for postal codes in Scotland) databases, which associate postal codes of residence with measures of deprivation determined from the UK National Census [20]. The merits of using (S)IMD measures of deprivation was discussed recently [21]; in summary, they attempt a measure of the overall deprivation experienced by people living in an area by combining 38 indicators across seven domains (income; employment; health and disability; education, skills, and training; barriers to housing and other services; crime; living environment), weighting in particular income and employment. Deciles of rank deprivation were analyzed in preference to raw scores to reduce the number of levels for comparisons and mitigate any effects of the slightly different weighting used in deriving IMD and SIMD scores. Focus on the extremes of the deprivation range was achieved by further consolidation of the rank scores into three levels as described in Table 1.

Center factors were defined to capture possible influences from the centers providing renal services to patients. Centers were classified according to the date RPV was first offered to patients, the proportion of the Renal Replacement Therapy (RRT) population recruited to use RPV, and their provision or not of extra support to patients registering with RPV for the first time (assisted start).

**Table 1:** Patient factors.

Patient factors			Notes
Age in years		<18, 18-34, 35-54, 55-74, >75	
Gender		M/F	
**Treatment**			
		Hospital HD^a^	Includes most dependent patients
		Home HD/PD^b^	
		Transplant	
		Not on RRT^c^	
**Deprivation** ^d^			
	By decile	1-10	1 is most deprived
	**By three groups**		
		High	Deciles 1 and 2
		Middle	Deciles 3-8
		Low	Deciles 9 and 10
Access log data		Dates and times of every logon	
Blood test results		Sample dates and values	
Center		Unit code	UK renal registry code of treatment center

^a^HD: hemodialysis.

^b^PD: peritoneal dialysis.

^c^RRT: renal replacement therapy, so HD, PD, or transplant.

^d^Deprivation measures: see text.

### Classification of Users by Logon Activity

**Figure 1:**
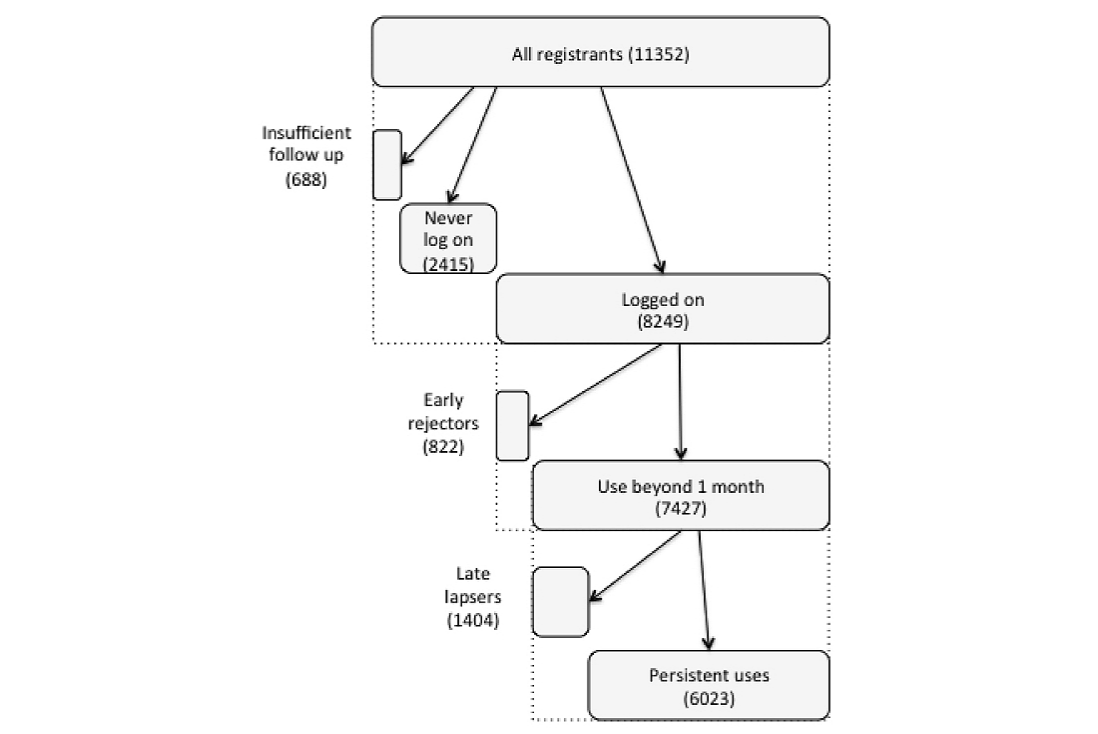
Classification of RPV users by logon analysis.

Figure 1 shows the approach taken to classify registrants according to their completion of the initial logon and password change procedures, their making logons during the first month post-registration through making logons right up to the time the RPV census was taken (persistent users). Registrants were classified into the following groups: (1) insufficient interval of follow up- patients enrolled within 6 months of the RPV census date were excluded from classification on the basis of logon activity as insufficient time had passed to determine any pattern, (2) patients that never log on, (3) early lapsers, that is, patients who log on only during the first month post registration, (4) late lapsers, that is, patients who at the time of census had not logged on for 6 months and for whom there had been at least 2 sets of blood result uploads (patients were therefore not deceased and had some incentive to log on), and (5) persistent users, that is, all other patients.

For some studies, patients were also classified by treatment in order to enable comparison with an appropriate overall UK population. This enabled comparison of adult RPV registrants in receipt of renal replacement therapy with the overall UK adult population in receipt of renal replacement therapy at the time of the RPV census as recorded at the UK Renal Registry report 2009 [22].

### Exploration of Factors Possibly Associated With Registration and Usage

Statistical analysis used the R framework [19]. Logistic regression used the glm program of the R stats package. The approach was to initially include all the factors listed above, then iteratively remove less significant factors and test the reduction in variance explained by the simplified model (analysis of variance) to decide their removal.

### Persistence of Use

Survival (of RPV use) analysis employed the survplot program [23]. Use was viewed as beginning on date of first logon and ending on date of last logon. Use durations were right-censored where last logon dates were judged consistent with continuing use of the system: specifically, last recorded logon was either within 6 months of the RPV census date, or earlier but not succeeded by at least 2 results events (suggesting infrequent follow-up). The last provision also right-censored patients who had been users of RPV up to death.

### Analysis of Logon Activity

The overall intensity of use was computed by counting, for each patient who completed at least 1 logon, the total number of logons made and dividing by the number of months between registration and census to compute logons per month. The distribution of the logarithm of logon activity across the patient population was approximately normal (see Results), enabling patients to be classified by the quartile (Q1-Q4) that their individual log (logons per month) fell in the overall log (logons per month) distribution.

The timing of logon events in relation to test results was explored first with a plot devised to show the activity of individual patients as series of colored dots along a horizontal timeline. The activity of approximately 100 patients could be plotted without obscuring individual patient events, so plots show random selections of patients from appropriate subgroups ordered by the duration of logon activity. The overall tendency of logon events to occur close in time to test result events was explored by computing for every logon event the interval to the closest result event and constructing a histogram to show the proportion of logons by interval to closest test date. Significance was assessed by comparing histograms made with randomly shuffled logon times or test times.

## Results

### Which Patients Register to See Their Records?

**Figure 2:**
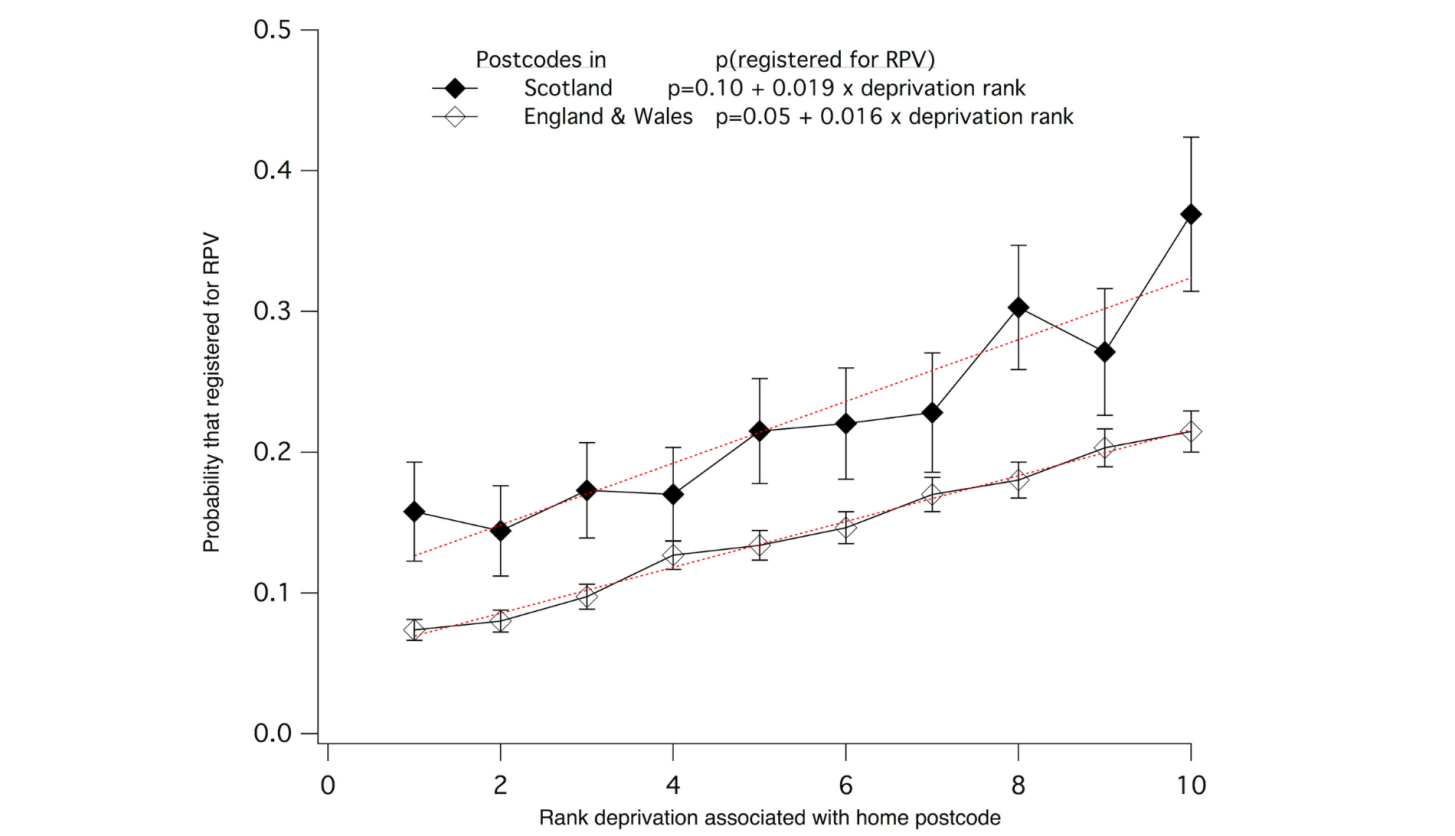
RPV registration by patient deprivation. The proportion of adult RRT patients registered for RPV is shown by patient rank deprivation (1-10, 10 is least deprived) for patients with postal codes in Scotland (filled diamonds) or England & Wales (open diamonds).

At the time of the RPV census, 11,352 patients had been registered from 37 of the United Kingdom’s 73 adult centers. The characteristics of the 11,352 registrants are shown in Table 2. Their age and sex distribution broadly parallels the age range and male propensity of renal disease [22]. To assess factors that might be associated with registration for RPV, the characteristics of registrants in receipt of renal replacement therapies (n=6646) was compared with that of all UK patients in receipt of renal replacement therapies (N=44,649). Overall, 14.8% of UK adult RRT patients were registered with RPV, but there were significant differences in the proportion registered by age, treatment, and deprivation (Table 3). The proportion registered in the 18-34 and 35-54 age groups were almost twice that of the over 75 age group, and the proportion among patients with transplants or on home-based dialysis (peritoneal dialysis or home hemodialysis) was about 70% greater than of that of hospital hemodialysis patients. The proportion registered increased almost linearly with increasing rank deprivation score (ie, with lower deprivation) (Figure 2). Patients residing at postal codes associated with the lowest levels of deprivation (rank 10) were 2.4 (England and Wales) to 3.2 (Scotland) times more likely to be registered for RPV than those at postal codes associated with the highest deprivation (rank 1). There were no significant differences by gender.

**Table 2:** Description of the entire RPV-registered population (N=11,352).

Characteristic		Proportion, %
**Age in years**		
	<18	1.6
	18-34	11.6
	35-54	37.9
	55-74	38.9
	>75	10.0
**Gender**		
	Male	59.9
**Treatment**		
	Hospital HD	19.4
	Home HD/PD	6.7
	Transplant	32.8
	Not on RRT	41.0

**Table 3:** Description of the RRT-dependent subgroup (n=6646) of the RPV-registered population compared with the overall UK RRT population by age, gender, and treatment (N=44,649)^a^.

Characteristic		Proportion registered for RPV of all UK RRT patients, %	*P* value
**Age in years**			
	18-34	16.1	*P*<.001
	35-54	16.6	
	55-74	14.8	
	>75	8.4	
**Gender**			
	Male	15	Not significant
	Female	14.6	
**Treatment**			
	Hospital HD	10.5	*P*<.001
	PD	17.7	
	Transplant	16.6	
**Overall**			
		14.8	

^a^Significance of comparisons estimated by Pearson’s chi-square test statistic.

**Figure 3:**
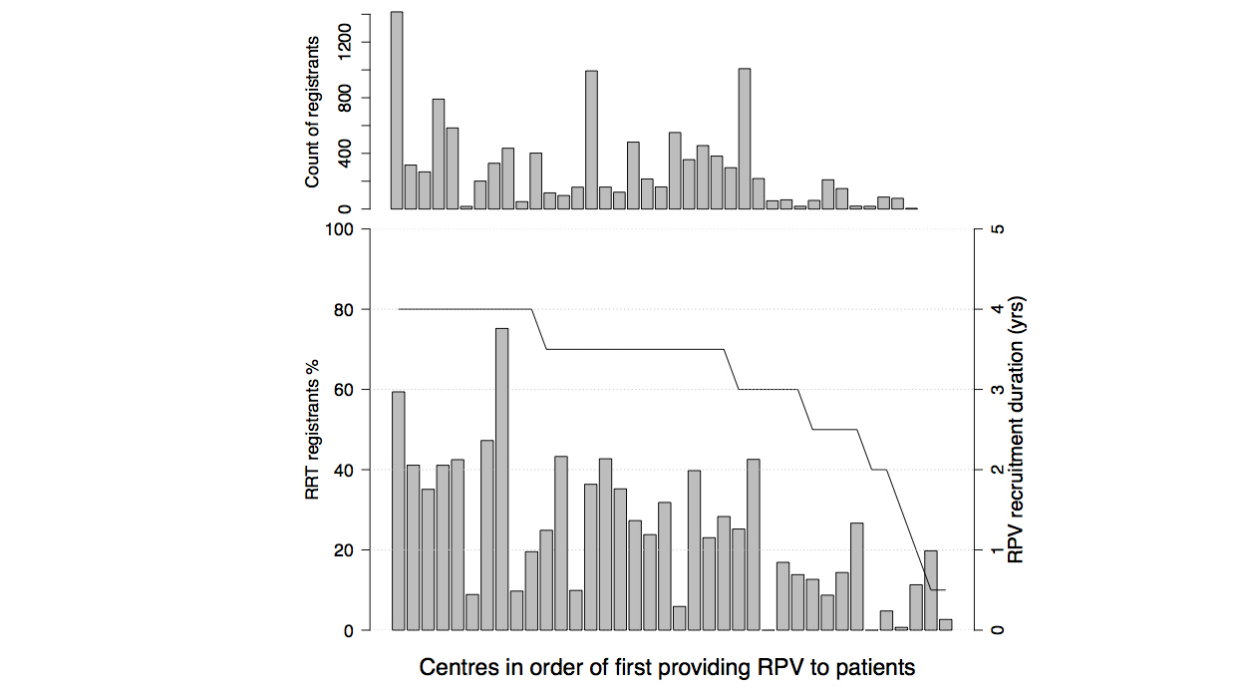
RPV registration by center. Number of registrants (top chart) and proportion (%) of available RRT patients registered for RPV is shown by renal center, ordered by the start date of patient enrollment. The duration of active RPV enrollment is superimposed on the bottom chart; duration ranged from 0.5-4 years.

The penetration (proportion of adult RRT patients registered) by center ranged from 6-75% (median 32) for centers enrolling at least 6 months. The variation was only in part explained by differences in the duration of recruitment effort (6-48 months) (Figure 3), so very likely it was influenced by centers’ recruitment practices. One very significant difference in recruitment practice was identified (see below), but formal definition of the influential factors is a goal of ongoing investigation. It is noteworthy, however, that many of the centers with higher recruitment have enthusiastic local proponents of RPV within their clinical teams.

### Completing First Logon

**Figure 4:**
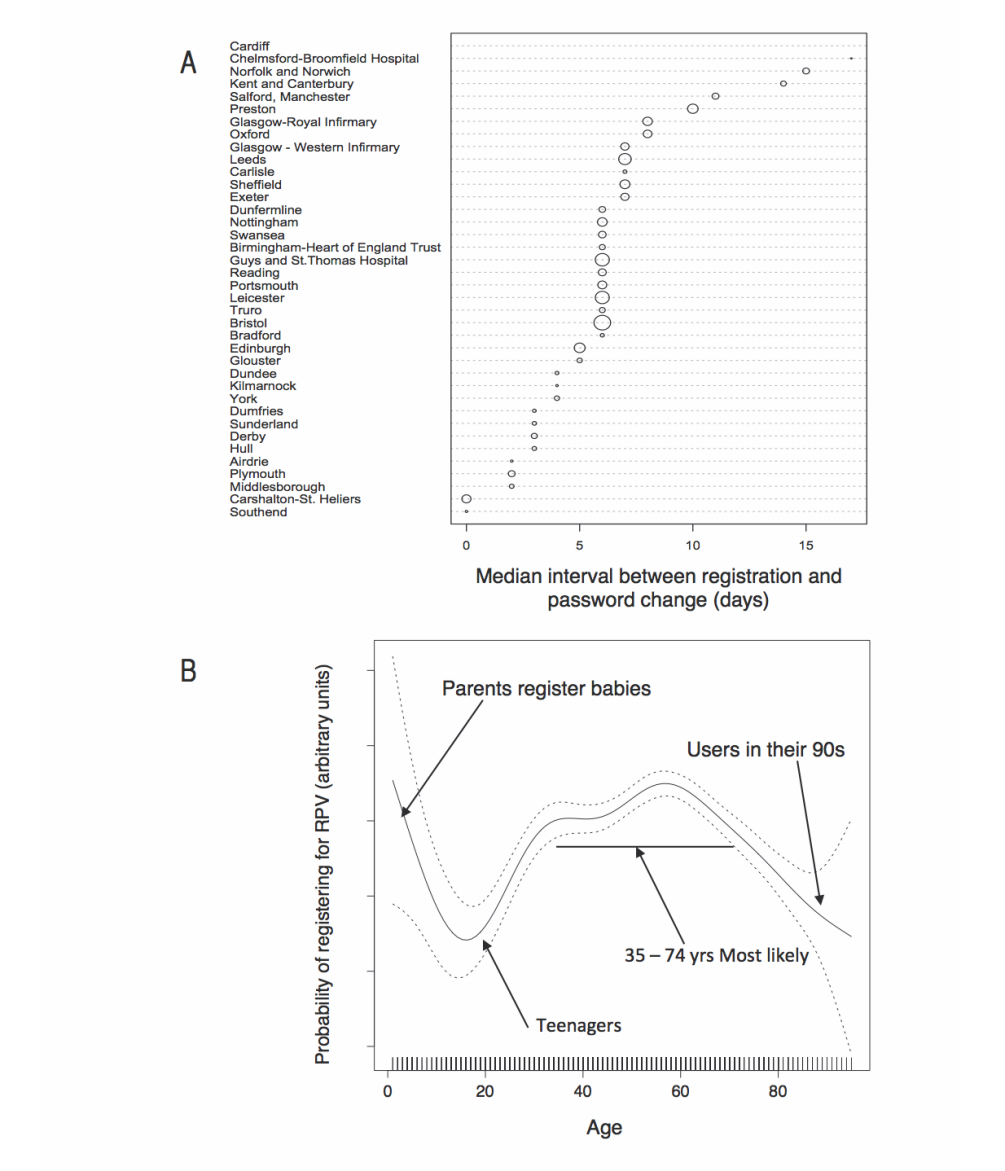
Interval from registration and likelihood of completing first logon. A: the median interval in days between registration and initial patient logon by renal center. The size of markers is proportional to total number of registrants at each center. Two centers are remarkable for completing the process within a day for most patients (87% of patients at the larger center). B: Probability of completing first logon by age. A logistic (completed first logon = T/F) general additive model (non-parametric) was constructed using the mgcv package to model the likelihood of patients completing first logon by age at time of registration without assumption as to the shape of any relationship.

About one-fifth (23.20%, 2634/11,352) of registered patients never logged in despite having signed up and having been sent details. Logistic regression indicated that the strongest registrant-specific effects (up to 2-fold) were age and treatment group (Table 4). Registrants in middle age were more likely to complete first logon than younger (<34) and older (>75) registrants, and registrants with a transplant were more likely to log on than patients on hemodialysis. The complex influence of age was further studied avoiding arbitrary age grouping by using a general additive model and non-parametric smoother (Figure 4B). This revealed that the small number of very young registrants were the most likely of all to complete first logon, presumably represented by their parents.

The effect of deprivation was also very significant. Registrants from addresses associated with greatest deprivation were more likely to not complete first logon (OR 1.24, 95% CI 1.08-1.42), whereas registrants with addresses associated with low levels of deprivation were less likely to not complete first logon (OR 0.79, 95% CI 0.65-0.97), both compared with registrants of middle rank deprivation, indicating a negative influence of deprivation beyond that on becoming registered, as described in other chronic disease populations [11,12].

Patients at centers that began offering RPV in the 2 years prior to the census were twice as likely to not complete first logon (OR 2.04, 95% CI 1.41-2.93) as patients at centers that had been offering RPV for more than 2 years, which seemed greater than the lag to be expected for recently enrolled centers to build up recruitment. This is a subject of continuing investigation.

**Table 4:** Odds of not persisting with RPV use at proposed hurdles^a,b^.

		Odds not completing initial logon (N=9552), OR (CI)^c^	Odds lapse early (N=822 vs 7427), OR (CI)	Odds lapse late (N=1401 vs 6023), OR (CI)
**Age (compared with age 35-54)**				
	<18	1.81 (1.27-2.55)	1.02 (NS)	0.76 (NS)
	18-34	1.32 (1.12-1.56)	1.21 (NS)	1.03 (NS)
	55-75	0.98 (NS)	1.02 (NS)	1.11 (NS)
	>75	1.41 (1.19-1.66)	1.46 (1.12-1.90)	1.58 (1.27-1.96)
**Treatment (compared with hospital HD)**				
	Pre RRT	1.0 (NS)	0.83 (NS)	0.37 (0.31-0.44)
	Home HD/PD	0.88 (NS)	0.96 (NS)	0.68 (0.53-0.86)
	Transplant	0.60 (0.52-0.69)	0.61 (0.49-0.75)	0.45 (0.38-0.52)
**Deprivation (compared with middle)**				
	Greatest	1.24 (1.08-1.42)	1.73 (1.40-2.13)	—
	Least	0.79 (0.70-0.89)	0.79 (0.65-0.96)	—
Unit offering RPV <2 years		2.04 (1.41-2.93)	—	—
Unit registration rate^d^ in Q3 (Q2 also significant)		0.79 (0.65-0.97)	—	—
Unit offering routine assisted start		0.31 (0.21-0.46)	—	—

^a^Summaries of models obtained by logistic regression for the likelihood that patients choose not to persist with use of RPV at the three decision points: (1) choosing not to complete first logon, (2) having made an initial logon choosing not to logon on again beyond 1 month (lapse early), and (3) discontinuing logons at some later time (lapse late).

^b^Odds ratios are shown for the influential factors followed in brackets with 95% confidence intervals or NS if the interval spans 1.0. Factors marked with a dash (—) were removed because of insignificant effects in the indicated model. Gender had insignificant effects in all models.

^c^Analysis restricted to the 9552 (of 11,352) registrants with complete data. The major reason for exclusion was missing treatment type as a result of this parameter not being recorded by one major center (1009 registrants). Alternative analysis including these 1009 registrants and excluding treatment as a factor did not for any other factor change the assignment of significance and only slightly altered the ORs.

^d^Centers were grouped in quartiles of percentage enrollment of RRT patients as a measure of a center’s effectiveness in recruiting patients to RPV.

### Interval Between Registration and First Patient Logon

The interval between registration and first logon was investigated because it was thought likely to be a marker of patient enthusiasm. However, the interval was dominantly influenced by center (Figure 4A). Most striking was the very short interval (<1 day) at two centers (Carshalton and Southend) leading to the discovery that these centers had elected to have an administrator help patients complete their first logon immediately after registering, in contrast to the usual practice of sending logon instructions by mail. Unsurprisingly, patients of these 2 centers were unlikely to not complete first logon (OR 0.31, 95% CI 0.21-0.46) when compared to patients of all other centers. More importantly, this practice also influenced subsequent logon behaviors (see below).

### Persistence of Use After First Logon

There was substantial variation in both the frequency and persistence of RPV logon activity subsequent to first logon. As some registrants had been registered only shortly before the RPV census such that their logon activity was too brief to be assessed, analysis of subsequent logon activity was restricted to the 8249 registrants who completed first logon more than 3 months prior to the census. Three broad patterns were distinguished (Figure 1); 822/8249 (9.96%) made no logon beyond the first month suggesting they were disinclined to continue engagement (early lapse). Of the 8249 (90%) who made use of RPV beyond 1 month, most (6023/8249, 73.01%) made continued use up to the census date (persistent users), but 1404 (16.98%) were judged to have lapsed (perhaps wrongly, see Discussion) in their use of RPV because at least 6 months had passed and at least 2 sets of test results had been uploaded since last logon (continued arrival of test results confirming that they were still alive). The 8249 registrants who made at least one logon went on to make a total of 1-914 (median 14, mean 42.7) logons over 0-42 months. Among those classified as lapsing and persistent users, the median number of logons was 4 and 26 respectively.

Factors that might influence subsequent logon behavior were investigated first by logistic regression. Early lapse was associated with age over 75 and greater deprivation and was less likely in transplant recipients (Table 4). Late lapsing was associated with age over 75 and treatment by hospital hemodialysis. Interestingly, deprivation was not a significant factor in this cohort of established RPV users.

**Figure 5:**
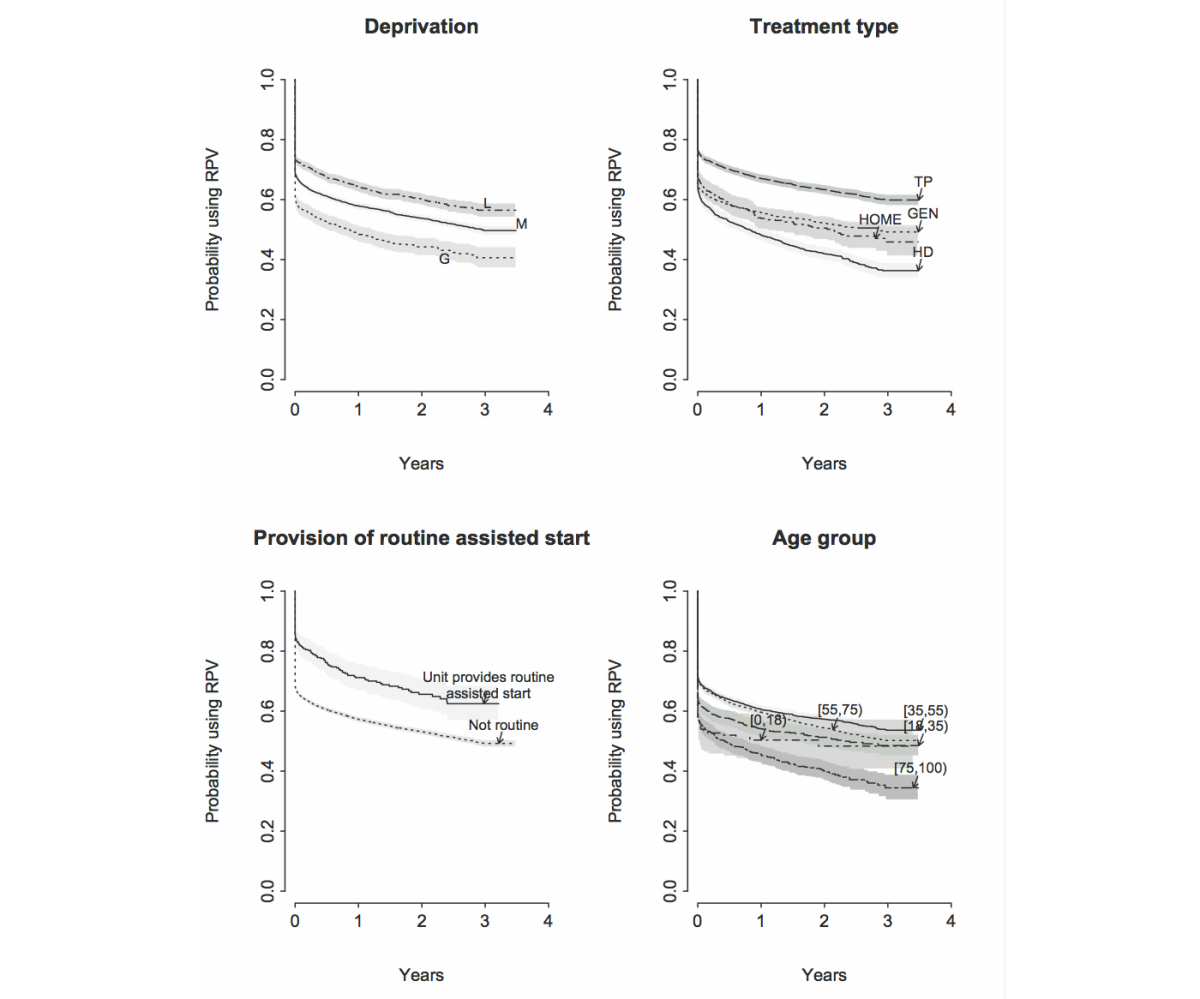
Probability of persisting use of RPV at intervals after registration shown as survival (of RPV use) classified as shown. Shading indicates 95% confidence limits.

Survival analysis was used to better understand lapses in use of RPV over time, plotting probability of continuing use by time for users partitioned by relevant factors (Figure 5). Overall, the probability of continuing use was 0.61 (95% CI 0.602-0.621) at 6 months falling gradually by about 0.04 per year (6 months 0.61, 12 months 0.58, 18 months 0.56, 24 months 0.54, 30 months 0.52). Interestingly, while greater deprivation was associated with substantially reduced probability of continuing RPV use at 6 months, as might be expected [14], the subsequent rates of attrition were similar in all three deprivation groups. In contrast, the greater early attrition in the elderly and hemodialysis treated was followed by a continuing higher rate of attrition than in the young and those with transplants.

The substantially greater early use observed in users at centers offering assisted starts was succeeded by a slightly greater rate of attrition out to 2.5 years leading to some convergence of the survival curves, but the beneficial influence remained highly significant.

### Patterns of Use Over Time and in Relation to Test Results

An immediate peak of very frequent logons was observed, which settled to a slowly declining average rate of logons. This concealed a highly variable rate of logons for individual patients, with periods of intense activity associated with periods of more frequent test results. These periods are likely to be times of medical uncertainty; for instance, some could be around transplantation or other hospital admissions.

**Figure 6:**
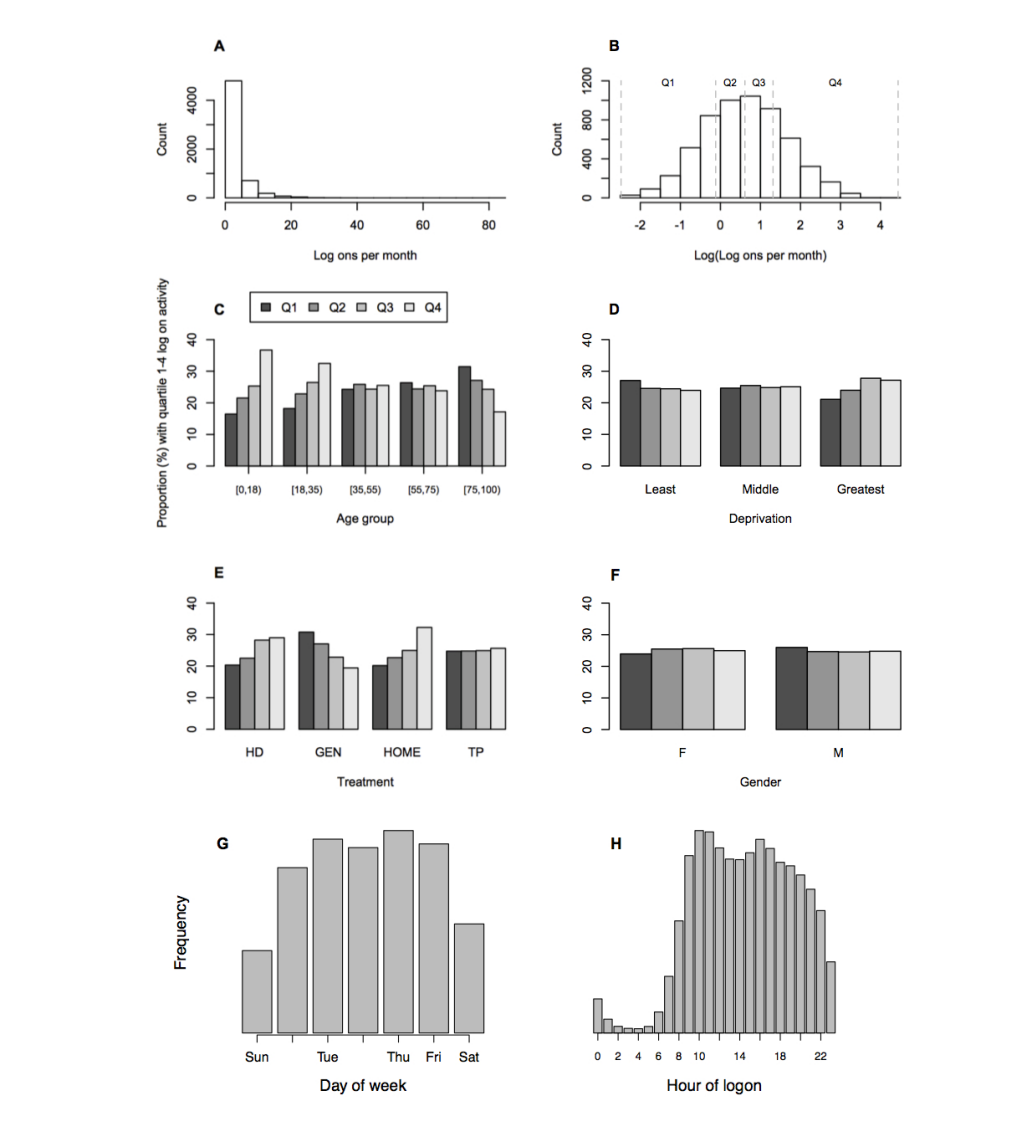
Logon activity. A-F: Patients classified by quartile of log (count of logons per month) (defined in panels A and B as described in the Methods), and comparisons made of the partitioning of Q1-Q4 activity patients by the factors shown. The even spread by gender contrasts with that by age (C) and treatment type (E). G-H: Histograms of counts of logons made by day of week (G) and time of day (H).

Frequency of use overall was assessed from the number of logons made by patients divided by the interval between first and last logon. This analysis was limited to the 5808 users who had made at least three logons over at least 3 months after first logon; 87.55% (5085/5808) were persistent users and 12.45% (723/5808) late lapsers. The median number of logons per month was 1.8 (2 for persistent users, 0.94 for lapsers) with a markedly skewed distribution (range 0.08-84) (Figure 6A). Subsequent comparisons were made of the natural logarithm of logons per month, which was distributed near normally (Figure 6B), categorized by quartile (Q1-Q4 where Q4 exhibits the heaviest use). The proportion of male and female users with Q1-Q4 logon frequency was very even, but with respect to age, users in the two younger age groups had a predominance of higher frequency logon activity (Q3 and Q4) and users in the oldest age group had a predominance of lower level activity (Q1 and Q2) (Figure 6C). In contrast with the negative influence of greater deprivation on registration and initial logon, within this cohort of established users, patients with addresses associated with greater deprivation exhibited a slight predominance of higher logon activity (Figure 6D). With respect to treatment type (Figure 6E), users on home-based dialysis included the highest proportion of higher-level users, which suggests patient self-monitoring. Higher-level use was also prevalent among users on hospital hemodialysis, a population that in most centers has frequent blood test monitoring (commonly monthly), providing frequent incentive to check results.

The patients exhibiting the most frequent logon activity (top 5%) were also analyzed separately. None of the factors available was associated with this extreme pattern of use.

Most logon events occurred on weekdays and between 08:00-22:00; in particular, Sunday logon was not popular (Figure 6G and H). No differences in the times of logon events was observed by age, sex, treatment group, or deprivation.

**Figure 7:**
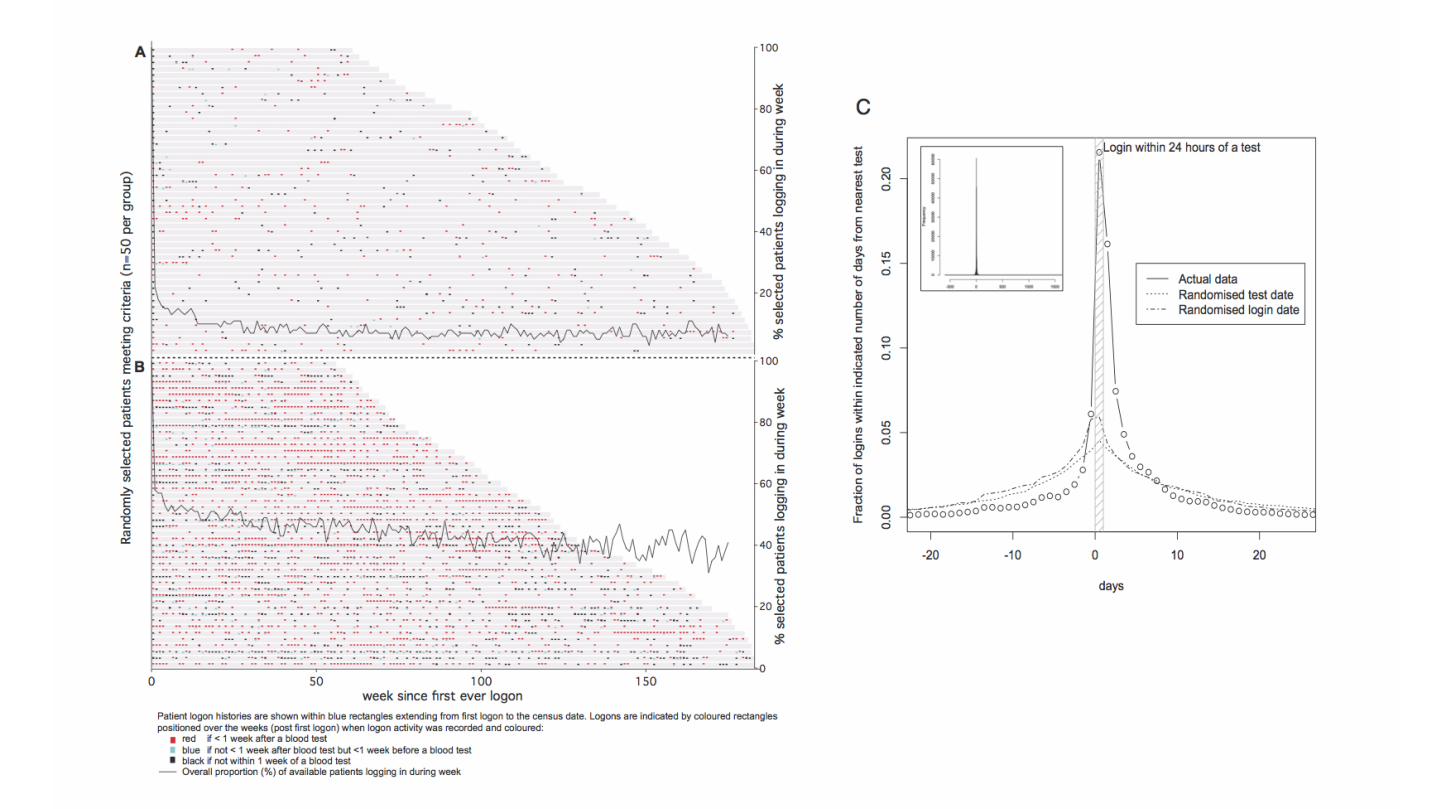
Relationship between times of patient logons and blood test results. A, B: Individual logon events shown by interval (in weeks) since first logon for randomly selected lower (A) and higher (B) frequency transplant recipient users where each selected patient’s activity is depicted by a horizontal series of colored dots. Most logon events are red indicating they occurred less than 1 week after a new blood result event. Superimposed is the proportion (% on the right hand axis) of patients in the respective activity groups that log in each week post first logon. C: Histogram (1 day bins) showing the predominance of logons occurring 1-3 days after tests is strikingly different from that observed with randomly shuffled data.

The time of logons to RPV was also examined in relation to the most proximate blood tests because surveys had suggested that accessing blood test results was a major reason for using RPV. First logon events were plotted against interval since first logon to visualize the patterns of logon activity. Examples are shown in Figure 7. There was clear variation in patient logon activity with the most intense activity typically occurring over short periods during which it is likely clinical circumstances increased the need for frequent updates. Most events occurred in the week after new blood test results became available when logons were likely performed to get the results. This was investigated further by determining for all logon events the interval from the most proximate new test result date: 75% of logons occurred within 2 weeks of new results becoming available (Figure 7C). The most likely interval was 0-1 day (21.6% of all logons), followed by 1-2 and 2-3 days (0-3 together 45% of logons), but surprisingly the 4^th^ most common interval was the 24 hours before blood tests were taken, when results could not possibly be available. Indeed, 10.8% of all logons occurred in the 3 days leading up to a blood test, suggesting that registrants are reviewing their results before a clinical encounter.

## Discussion

### High Rate of Persistent Use

It is striking and encouraging for advocates of patient-accessible EHRs that over half of registrants became persistent users (median of 2 logons per month) with only a small proportion (about 4% per year) discontinuing regular use out to 3.5 years. More persistent use was expected as CKD is a chronic condition, but the rate is much higher than the 26-33% reported in other studies that have assessed use by patients with chronic diseases (eg, [7,8]). RPV was developed by clinicians with close involvement of patients and iteratively improved over years to meet patients’ needs, but other systems make similar claims. Clearly RPV is providing services with an enduring appeal to users, and the results suggest that key among these is the timely provision of blood test results.

### Access to Timely Blood Test Results is a Key Driver to Renal PatientView Use

Blood test results are important in the continuing management of CKD informing, for example, the need for and effectiveness of dietary prescriptions as well as the progression of kidney failure or transplant performance, and many patients take a close interest encouraged by their doctors. Provision of timely blood test results was a core design goal for RPV and was identified as a key attraction for users in a small survey [6]. The current data provide strong support for the importance of this service demonstrating that 45% of all logons occur in the 3 days after a blood test was taken, presumably to look up the results. As well as indicating that access to results is what patients want, the finding is also indicative of a patient group that is engaged with their treatment—why otherwise look up the results and try to interpret their meaning? Further support comes from the observation that almost 11% of logons took place shortly before clinical encounters, suggestive of engaged patients wishing to be current with their results before potential opportunities to discuss their management with clinicians. Certainly clinicians report that consultations with some patients have changed as a result of patients’ access to RPV. An important question for ongoing study is the influence, if any, on the achievement of treatment goals as a result of greater patient involvement.

### Frequency and Timing of Use

Analyzed as a group, after an early phase of frequent logons, during which patients may be gaining familiarity with the system and learning from the information links provided, the frequency of logons by persistent users settles to a median of 2 logons per month with most logons during usual waking hours and the working week (Figure 6F, G). This is approximately twice the rate at which new results become available for most patients. Analysis at the individual patient level reveals considerable variation including some patients making very frequent logons (several per day), but in most cases this was observed to be short lived (a few weeks or months), possibly reflecting periods of greater concern, such as around the time of transplantation. Greater understanding will require a formal qualitative analysis, which is underway.

### Initial Patient Support Increases Persistent Patient Use

It is well recognized that initial authentication of new users is a substantial barrier for some patients. In the current data, 20% of patients never made a single logon despite having completed a registration form, returned it to the center administration, and become registered. The first logon procedure for new RPV users is explained in the registration letter and requires navigation of an internet browser to the RPV website, entering the temporary credentials and creating a new password. An important observation from the current data is that providing additional support targeted just at this first logon hurdle has a profound and long-lasting positive influence on patient use. Two centers adopted the practice of providing initial support in the form of face-to-face or over-the-phone guidance through the first logon procedure, with about a 20% increase in the probability of patients’ continuing use of RPV at 3 years (Figure 5, bottom left). Most centers give little to no active instruction at present. It may be that sufficient support could be provided more broadly without unmanageable cost implications. This will become clearer if the practice becomes more widespread as is being encouraged by the current guidance to units providing RPV [24]. It must be noted that this was a serendipitous retrospective finding, so further study is required. However, the results support a compelling case to better understand the practical issues faced by patients in accessing online records and the effectiveness of support strategies, both of which we are addressing in current research.

### Mixed Inclusiveness of Access

Inclusiveness is a concern in digital service provision [11,12,15,25,26]. It is remarkable that we observed no sex difference in any of the measures studied, as most studies show that women are more likely to seek medical advice and information both in person and online. Age was influential—RPV users are slightly younger than non-users (3 years on average)—but this small difference may be reducing as it was 7 years in an earlier survey of RPV users, admittedly comparing results obtained by different methods (survey versus log data). It is possible that logon sharing decreased sex and age differences. RPV encourages patients to share logons and many do. Up to 40% have shared logons in small surveys, and some are dependent on others to log on for them.

Level of deprivation was strongly associated with RPV registration and initial use. This is a new observation with regard to RPV but was anticipated from the results of many studies of access to EHRs and health care information on the Internet [11,12,25,26]. However the frequency of use of RPV is actually higher and the rate of drop-out after first logon is lower among users of greater deprivation. The explanation for this difference could be trivial (deprivation by postal code is imperfect) or very significant for best design of approaches to recruitment and initial support that are effective across the patient population. Digital exclusion is identified by the Department of Health in the United Kingdom as an area of high current concern [27]. Follow-up studies will address this and also determine whether the deprivation gradient is reducing with time (as it seems to be for age). Other means of access to RPV (digital TV, smartphones) are also under consideration.

### Conclusions

Renal PatientView attracts strong and sustained use by many renal patients in the United Kingdom. The current data indicate that prevalent center and patient factors underlie considerable variation in RPV uptake and use. These require further study, but it is already clear that centers should consider reviewing their approach to recruiting and supporting patients and possibly identify patients that are not using RPV post-registration to offer targeted help.

## Abbreviations

CKD: chronic kidney disease (irreversible, often progressive kidney damage)
HD: hemodialysis
EHR: electronic health record
PD: peritoneal dialysis
RPV: Renal PatientView
RRT: renal replacement therapy (such as dialysis or kidney transplant)
